# Cyber Risks Prediction and Analysis in Medical Emergency Equipment for Situational Awareness

**DOI:** 10.3390/s21165325

**Published:** 2021-08-06

**Authors:** George Burke, Neetesh Saxena

**Affiliations:** School of Computer Science and Informatics, Cardiff University, Cardiff CF10 3AT, UK; burkegj@cardiff.ac.uk

**Keywords:** cyber risks, situational awareness, manipulation attack, healthcare

## Abstract

In light of the COVID-19 pandemic, the Medicines and Healthcare products Regulatory Agency administered the standards for producing a Rapidly Manufactured Ventilator System (RMVS) free of charge due to the United Kingdom’s shortfall of ventilator systems throughout health centers. The standards delineate the minimum requirements in which a Rapidly Manufactured Ventilator System must encompass to be admissible for usage within hospitals. This work commences by evaluating the standards provided by the government to identify any potential security vulnerabilities that may arise due to the succinct development standards provided by the MHRA. This research investigates what cyber considerations are taken to safeguard a patient’s health and medical data to improve situational awareness. A tool for a remotely accessible, low-cost ventilator system is developed to reveal what a malicious actor may be able to inflict on a modern ventilator and its adverse impact.

## 1. Introduction

The increasing connectivity of modern medical devices to computer networks and the convergence of technologies are steadily exposing vulnerabilities within the devices and the software applications they employ. The medical device companies must intend for front-line usage explicitly consider all aspects of the devices’ security throughout their life cycle. This includes the device’s design, procurement, monitoring/auditing, and operation [1]. Health trusts across the United Kingdom employ medical devices to perform life-critical tasks on a patient and are highly dependent on the systems running uninterrupted. These systems perform a wide range of activities in which a human may find challenging to accurately emulate. An example of this would be utilizing a ventilation system to provide a specific respiratory rate (number of breaths per minute) to a patient suffering from a respiratory-related illness. It is crucial the companies that are manufacturing these devices highly dependent upon medical devices incorporate an extensive level of security into medical device systems to prevent malicious actors interfering with how the system functions as otherwise, major repercussions can transpire.

Healthcare organizations are particularly vulnerable and targeted by cyber threats as they possess high levels of information of high monetary and intelligence value to cyber attackers and nation-state actors. This is typically the patient’s data and privacy that is at risk, and potentially their health. The UK’s NHS is no stranger to cyber-attacks—falling victim to a ransomware attack in May 2017 known famously as the WannaCry attack [2]. This attack rendered medical devices including computers, MRI scanners, blood-storage refrigerators, and theatre equipment inoperative. This attack was feasible due to the outdated Windows XP operating system being used on thousands of computers within particular trusts throughout the nation. The Windows XP operating system contained major security flaws which malicious actors were able to successfully exploit, costing the NHS £92 million in disruption to services and IT upgrades [3]. With this in mind, it is essential that cyber security is more directly integrated into the fabric of healthcare and must essentially be viewed as an organizational asset that is seen as customary and mission critical as hygiene standards and patient safety procedures have become with quality care [4].

***Cyber Security Issue:*** An unprecedented epidemic of pneumonia of unknown etiology emerged in December 2019. A coronavirus was identified as the causative agent, and this was deemed COVID-19 by the World Health Organization (WHO). COVID-19 is caused due to a beta coronavirus named SARS-CoV-2 which targets a human’s lower respiratory tract and manifests as pneumonia [5]. A crisis such as COVID-19 creates vulnerabilities. Since the introduction of the pandemic, the WHO has recorded an influx of cyber-attacks on businesses [6]. Malicious attackers presuppose that amidst a crisis, critical services such as hospitals cannot afford to be locked out of systems and assume that a ransom would rather be paid. On the 13 of March 2020, Czech Republic’s second-biggest hospital fell victim to a cyber-attack. The attack temporarily breached security and successfully limited the operations of computer systems throughout the hospital. The hospital has not disclosed the nature of the security attack, although, the incident was regarded severe enough to postpone urgent surgical interventions and reroute patients to an alternative nearby hospital [7]. Cyber-attackers and state-backed hackers will take full advantage of a pandemic to inaugurate attacks, invoking fear and uncertainty in a time of susceptibility. A shortfall of mechanical ventilators and other medical equipment including PPE for staff was noticed throughout trusts and required attention if the nation aspired to conquer the crisis [8].

As medical devices become more connected and more technologically advanced, extra precautions must be taken regularly to identify modern cyber risks to the devices and to also highlight any security vulnerabilities in which the medical device may possess. On the 20 of March 2020, the United Kingdom’s Medicines and Healthcare products Regulatory Agency released the standards delineating the bare minimum requirements in which a Rapidly Manufactured Ventilator System (RMVS) must possess to be regarded fit for usage within hospitals during the current COVID-19 pandemic. A consortium of companies from the United Kingdom joined forces to rapidly produce medical ventilators to meet the growing needs of the nation. This group, including the likes of Microsoft, Ford, AIRBUS, and many other reputable companies labeled themselves the VentilatorChallengeUK consortium; guided by Dick Elsy, CEO of High-Value Manufacturing Catapult, a group of manufacturing research centers in the UK [9]. Many other companies across the nation have taken it upon themselves to construct their ventilator systems to assist with the pandemic demand. These relaxed specifications provided by the government delineate what secure software development standards need to be adhered to when constructing the core of a ventilator; however, no real consideration toward the cyber security of the Rapidly Manufactured Ventilator System is detailed. As a causative factor of COVID-19, cyber threats increased by six times their usual levels in April 2020 as cyber criminals leveraged on the global incident in an attempt to further their agendas [10]. This study explores the potential cyber security vulnerabilities associated with the RMVS model, highlights the modern cyber security threats to medical devices, and creates a networked environment where a medical device can be controlled to express the importance of stringent network security for medical devices.

This study intends on investigating the recent state of smart medical devices along with what cyber security considerations are implemented into medical devices to protect a patient’s safety and their corresponding medical data from cyber threats. To utilize low-cost microchips (NodeMCU and Raspberry Pi 4) to construct a budget simulation of a remotely accessible medical device. This device will be assigned to a LAN whereby authorized users can visit the address of the website, enter their credentials, and control the ventilator simulation from another location. To make use of a threat modeling methodology to exhibit any vulnerabilities associated with the budget simulation. The small-scale representation of a remotely accessible medical device could unveil vulnerabilities that may exist on newer, larger-scale medical devices. These medical devices boast comprehensive features and have been rapidly manufactured and deployed with minimal cyber threat consideration. To provide security mitigation strategies for low-cost, provisional medical device implementations. These mitigation strategies could include encrypting medical devices from point A (medical device) to point B (web server controller) to utilizing secure development practices.

Our work investigated United Kingdom standards for producing an RMVS, particularly, highlighting the key requirements on securing such systems and improving situational awareness. In summary, our work makes the following contributions:

- We provide an understanding and analyze the UK standards and requirements for RMVS from the cyber security point of view.
- Through our situational awareness-based approach, we show how an attack scenario would be different from the normal scenario dealing with malicious data to be sent over the network and its adverse impact by setting up new experiments and developing a tool.

The rest of the paper is organized as follows: Section 2 provides background and Section 3 discusses related works on cyber security issues around medical devices and communication networks and protocols, web-based telemedicine ventilators, cyber vulnerabilities, and security controls. Section 4 describes our approach and Section 5 presents experimental design and implementation along with our findings—normal scenario vs. attack scenario with its adverse impact. Finally, Section 6 concludes the paper and discusses future work.

## 2. Background

The increasing connectivity of medical devices to computer networks and the convergence of technological enhancements provide clinicians with cutting-edge systems that possess extremely beneficial and life-saving features. It is understood that implementing this functionality increases the risk of potential cyber-attacks as more avenues to exploit are present. The drastic impact this could pose on clinical care and the safety of patients is causing distress for healthcare businesses, regulators, and medical device manufacturers [1]. 

### 2.1. System Model

**Figure 1:**
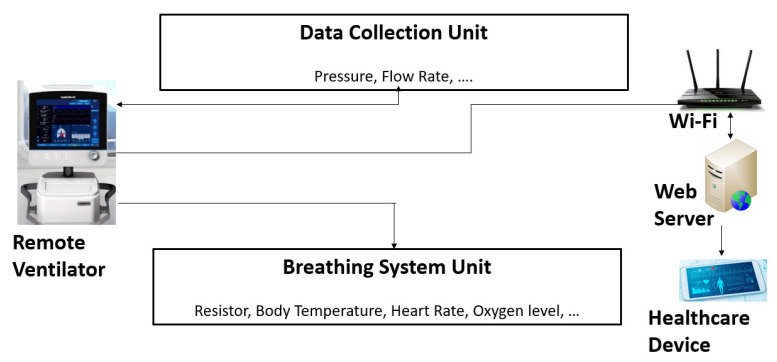
Remote ventilator system model.

As shown in Figure 1, the ventilator system model consists of a Cellular Ventilator Monitor (CVM) that was fixated to the patient’s ventilator along with a web server. The communication between the web server and the medical equipment like the ventilator is provided through a Wi-Fi connection for our testing work. This communication technology can be changed as the speed and reliable connection requirements with other networks such as 5G. The software prepares data for transmission and sends it to reside on the web server. The web application through devices such as smartphones or laptops enables clinicians to access this information through a web browser.

### 2.2. Medical Devices

A statement of distress was released by the FDA in 2019 discussing potentially serious cyber security pitfalls in some medical devices, potentially allowing hackers to control the devices from a remote location [11]. Regulators did not disclose what medical devices were included under this susceptible category; however, it is known that insulin pumps, pacemakers, ventilators, and MRI scanners are the target.

***InsulinPumps:*** In 2017, the Industrial Control Systems Cyber Emergency Response Team (ICS-CERT) acknowledged potential radio frequency vulnerabilities within insulin pumps. Due to the insulin pump segmented design, an attacker can compromise both the communications module and the therapeutic module of the pump. Two years later, Medtronic had to recall a large number of insulin pumps as a critical vulnerability was discovered that enabled remote attackers to modify the delivery of an insulin bolus dose [12]. An attacker had to option to repeatedly give a patient doses of insulin or override a patient’s attempt to give themselves insulin. This interference over wireless transmission allows cyber attackers to exploit devices without visible action and can potentially kill the insulin pump user. An array of brute-force techniques is used throughout the attack to listen to transferring communications and to successfully replicate transmissions to send the insulin pump unauthorized commands.

***MRI andCT Scanners:*** Both MRI and CT imaging devices include an image reconstruction component for rendering a scanned image based on raw data [13]. Imaging devices require network capability to transfer patient results to another system. Medical imaging devices typically use the DICOM protocol for this task. DICOM is a standard that handles, stores, prints, and transmits medical imaging information. An attacker could exploit the network functionality of the device to perform damaging attacks such as altering the results of a scan. This type of attack can go unnoticed if performed well. More sophisticated attacks are possible, such as relating scan results to the wrong patients or relating incorrect results to a patient that is being scanned. A persecutor would also have the capability to remove tumors from a scanned image or including a non-existing tumor in an image. The severity of performing these attacks is high and carries injurious consequences. A patient that falls victim to such a cyber-attack may require immediate attention where the smallest delay could be fatal. Imaging devices are also prone to ransomware attacks as encrypting the host controller would render the device offline. In addition, encrypting scan images in critical situations would put patients at serious risk [13].

***MedicalRecords:*** Stolen Electronic Healthcare Records (EHRs) are an extremely desired asset to an attacker due to their soaring demand on the darknet. EHRs contain extremely sensitive information (medical diagnoses, billing information, policy numbers, PII) that can be applied by fraudsters to file false insurance claims and purchase medical equipment, drugs, and fake IDs. It is known that EHRs hold a very high price tag and can sell for thousands, whereas credit card information sells for a fraction of the price. This is because medical records are more challenging to recover or terminate, whereas a credit card can be frozen and card fraud is detectable.

***Pacemakers and ImplantedDefibrillators:*** Implantable cardioverter defibrillators (ICDs) and cardiac Re-Synchronization Therapy Defibrillators (CRT-Ds) provide pacing for slow heart rhythms and electrical shocks or pacing to stop precariously fast heart rhythms. These devices are implanted under the skin in the upper chest area with connecting insulated wires called leads that connect to the heart [14]. The FDA revealed information concerning cyber security vulnerabilities associated with the application of the Conexus wireless telemetry protocol that is included as the communication method between Medtronic’s ICDs, CRT-Ds, home monitors, and clinic programmers. The Conexus protocol makes use of Radio Frequency (RF) signals for communication between devices. This enabled the device to remotely transmit data for remote monitoring, allowed clinicians to display information in real-time, and remote programming of the device. The Conexus protocol used does not include encryption of data, authentication, or authorization. These vulnerabilities, if exploited, could enable an attacker to access and manipulate the implantable device, home monitor, or clinic programmer. This type of attack has the potential to cause serious harm to the user of the device.

***IoMT and HospitalNetworks:*** Networked medical devices or Internet of Medical Things (IoMT) devices are particularly susceptible to cyber threats as a large portion of these devices were not intended to include network capability when devised. Over 70% of IoMT devices employ the unsupported Windows 7 operating system that is no longer supported and is difficult to patch [15]. Many network topologies used in hospitals and health facilities are in an uncontrollable state of flux as many IoT devices have unchecked access to internal networks without undertaking prior security checks. Thousands of these devices are portable and can be easily moved between different ward networks and off-campus sites without question. It is estimated that around 10 billion IoMT devices are currently connected to the global clinical ecosystem, with 50 more devices being connected every second, and 50 billion devices projected by 2028 [16]. Monitoring every device seems like an impossible task without employing an automated IoMT management solution.

A networking solution that may mitigate cyber-attacks to hospitals and health facilities is to adopt a zero-trust security policy. Employing this solution would limit access to sensitive information as verification would be required from all nodes attempting to obtain access to resources stored on the network regardless of whether the attempt to access the data was from an internal or external device. Zero-trust policies assist with limiting the reach of external attacks by suspending the potential propagation of infection to sensitive devices on the network [16]. Network segmentation is another architectural approach that divides a large network into child sub-nets. This approach would reduce the attack surface of the clinical network by confining communications between devices to only those that are essential to upholding critical medical services.

### 2.3. Medical Device Security and ISO/IEEE 11073

Modern studies on the security of interconnected medical devices used in hospitals include new attacks such as Ransomware and vulnerabilities reported by security researchers and consulting companies [17]. These reports have shown the existence of vulnerabilities in all types of hospital networks, third-party networks (e.g., pharmacies and laboratories), and unpatched medical devices that could provide a perpetrator with vital access points, potentially providing malicious actors with admission to abuse hospital networks through allowing unauthorized access to critical interconnected medical devices [18]. Once a hospital network is breached, an attacker can move laterally across devices within the hospital to steal user credentials, exploit services, and locate other vulnerable and unhatched devices [19]. The reality is that many hospitals even in developed countries still depend on outdated technology due to the IT sector in hospitals being understaffed and underfunded. Equipment like ventilators should employ a separate network with special restrictions on communication and authentication control [20]. Examples, where similar stringent network security is employed, is within other critical sectors such as the chemical, financial, and defense sectors.

All UK-based companies producing these rapidly manufactured ventilators were attempting to do so following the guidelines provided by the MHRA that includes minimal cyber considerations [21]. Attackers can potentially target advanced attacks, including Ransomware and Radio Frequency attacks, over equipment such as ventilators to get access to the system [22]. In addition to this, researchers and developers across the world are still tirelessly experimenting with new ways in which medical devices can be operated from respectable distances. Researchers in Poland devised a remotely accessible ventilator whereby an authorized clinician can monitor and control a ventilator from a remote location using a web server [23]. The ventilator dashboard can be accessed by locating a particular web address and entering in valid user credentials. For these machines to be cost-effective, cheaper readily available components were utilized to construct the makeshift ventilators.

The advancement in medical devices to include capabilities such as sensing, networking, and computing enables a more efficient workflow in health centers. However, cyber-attacks on these medical devices are a serious threat. One work outlines the recent upsurge of medical device vulnerabilities reported by both the CVE database and ICS-CERT [24]. Throughout 1999–2018 around 110,500 vulnerabilities and exposures were documented in the CVE database. Just 354 (0.3%) of these vulnerabilities were reported to affect interconnected medical devices. The ISO/IEEE 11073 Health informatics standards allow for communication to occur between medical devices and external computer systems. The standards provide information on automatic and detailed electronic data capture of patient vital signs information and device operational data [25]. The purpose of this is to facilitate efficient exchange of vital signs and medical device data in all health care environments as well as to enable real-time plug-and-play interoperability for medical devices. As detailed in the 11073 standards, the architectural decisions taken to create this plug-and-play capability includes creating a point-to-point connection between an “agent” and a “manager”. A transport agnostic and an object-orientated philosophy is applied to allow porting to new communications channels and to facilitate code re-use. Agents are self-describing which enables managers to easily understand the characteristics of any agent device. The architecture is extensible so that new types of pre-defined agents can be understood. The ASN.1 notation for defining data structures is utilized for representing data structures and messages to simplify the parsing of messages between devices. The agents in this model are any Personal Health Devices (PHD), whereas a manager is typically associated with small computers or smartphones with greater computing resources [25]. Bi-directional PHD that support multiple access levels over potentially untrusted transports with limited resources is an immense cyber security risk. The IEEE 11073 PHD standards family lacks detail on how to ensure the security of data exchange. It assumes that data exchange is secured by other means, for example, a secure transport channel. Various work groups have been looking into this to prevent unauthorized access, unauthorized modification, misuse, denial of service, or unauthorized use of any information that is stored on, access from, or transferred to and from a PHD. These standards determine messages that travel between agent and manager but not how those messages should be moved. The wireless protocols used for transport were the Bluetooth Health Device Profile, USB Personal Healthcare Device Class, and ZigBee Health Care Profile. It is believed that more technologies will be defined in the future.

### 2.4. Medical Device Communication Protocols

A large number of medical systems make use of Bluetooth technology to enable the transmission of data. This is due to its low power consumption, interference avoidance from other wireless devices, and small distance in which the signal is dispersed. With the appropriate software, any device can integrate Bluetooth compatibility to enable data transfer between systems. BlueTorrent, a P2P file-sharing application based on ubiquitous Bluetooth-enabled devices has been mentioned in previous studies to enable data transfer between medical devices [26]. This study attempts to explore the feasibility and effectiveness of utilizing ad hoc Bluetooth networks to transfer medical records from medical devices to a centralized medical database. Originally, created for sharing multimedia, BlueTorrent divides data into small chunks before transmission via single-hop data transfers. All data recorded by sensors is stored on a Bluetooth-enabled minicomputer possessed by a patient. Nodes can request missing chunks of data from neighboring medical devices to assemble files using BlueTorrent. Once a member of hospital staff is within the vicinity of a patient and has their Bluetooth device equipped, data transmission from the patient device to the staff device can occur over Bluetooth using BlueTorrent. The member of staff upon entering a designated location will upload the data to a centralized server for processing. If Bluetooth version 2.1 is applied to this architecture, a patient’s data should not be susceptible to MITM attacks as Bluetooth 2.1 includes Secure Simple Pairing (SSP) [27]. If access to a patient’s minicomputer through malware infection is achieved, stored data could be manipulated before it is transmitted to the staff device. The latter possibility can have serious consequences, including death. Moreover, if a compromised or malicious minicomputer can extend malicious content to the staff device, the hijacked staff device could be used to access other people’s sensitive data or manipulate data before it is uploaded to the centralized server.

## 3. Related Works

There are a vast number of studies that explore the transferal of medical device data through Bluetooth due to its convenience. Another study discusses the design and implementation of a Bluetooth 4.0-based heart rate monitor system using the iOS platform [28]. This allows a user to monitor their heart rate in real-time from an Apple device. In this example, the data from the heart rate sensor is contained locally in a sensor-to-device network and does not reside on an external server. There are many security vulnerabilities in frameworks like this. One of the simplest exploits to a user’s privacy is anyone within the vicinity of the Bluetooth range can connect to the sensor and view the real-time recordings. Another option for data communication is the Zigbee communication protocol. Zigbee is a low power, low data rate, and close proximity wireless communication protocol used for creating personal area ad hoc networks. Zigbee holds many similarities to Bluetooth and is widely considered for medical device data collection. Table 1 outlines the features in both Zigbee communication protocol and Bluetooth Low Energy version 4.2. Both are quite complimentary to each other. Some developers are finding that synergizing both protocols together can make for very strong personal and local area network devices.

### 3.1. Communication Protocols

One previous study utilized the Zigbee protocol to develop an end-to-end remote monitoring platform established on the IEEE11073 standard for PHD [29]. The Zigbee communication technology was introduced to express how health data could be transported using smart meter infrastructure. The smart meter, contained in a patient’s residence, receives data from sensors attached to a patient through the Zigbee communication protocol. These sensors are for monitoring the patient’s vital signs and detecting irregularities. The smart meter includes an uncommitted slot for transferring small packets of data transparently using power line communication to a data center in the local vicinity and then through any wide area network to a hospital.

In this study, Zigbee (Zigbee Health Care Profile) is considered over other communication protocols as it supports domains from both telehealth and telecare on a single wireless technology. The Health Device Profile (HDP) for both USB and Bluetooth (BT) only supports devices from the telehealth domain due to restraints on the range, power, and the number of devices that can be connected concurrently [29]. The Zigbee communication protocol makes use of AES for network layer encryption as well as application layer security to reinforce the exchange of network keys between a centralized server on a hospital network and an authenticated device joining the network. In this study, Zigbee was found to be reliable in all respects and provided extremely good performance as a wireless communication technology. A similar study further explores the advantages associated with storing medical device data centrally in electronic records through a Zigbee wireless sensor network [30]. The ventilator device has a Zigbee Medical Device Interface (MDI) node connected that acts as a serial to wireless bridge to enable the device to transmit data within the WPAN. Zigbee’s self-forming and self-healing mesh network architecture allows for data and controls messages to transfer from one node to another through varied paths. This is useful in hospital environments as interference from walls, people and general obstacles can prove a major issue. As soon as the MDI on the ventilator is powered on, the device automatically joins the WPAN and is acknowledged by the server. A clinician can then associate the ventilator device to a patient through the usage of the GUI client. Data sent to the server is stored in the Electronic Health Record (EHR) and is displayed on a GUI client when a patient is being examined. The ventilator data transmitted to the centralized server comprises the volume, time, ramp, and occlusion pressure readings. A ventilator has many parameters in comparison to other medical devices and because of this, slightly more bandwidth is required [30].

Another study discusses the amalgamation of both the Bluetooth and Zigbee communication protocols to develop robust communication between medical devices and telecommunication infrastructure [31]. The interface was designed to transmit medical data from vital sign medical devices to a centralized processing unit using a wireless network. This allows for medical data to be acquired, processed, and transferred to a centralized location. The network framework consists of two types of devices: MDIZ and MDIZB. The MDIZ is used for acquiring data from a medical device, processing the data, and transmitting it to through the usage of a Zigbee network. The MDIZB device receives the data from multiple MDIZs and transmits it to a PC using a Bluetooth network. In this network, Zigbee is applied due to its low power usage, simplicity, and ability to include a large number of devices per network. One downfall of Zigbee is the low data rate. Therefore, it is only used as an interface that is directly connected to the medical device through a single Zigbee module. Bluetooth has a higher data transfer rate but requires a larger amount of power. Due to this, Bluetooth is utilized as the next interface to the data processing devices to receive the data. The medical data is transmitted using both the Zigbee and Bluetooth protocols to reach its destination. The framework also supports detecting the existence of the device, handshaking, and error correction to establish a reliable connection and to mitigate any potential disturbances [31]. The results of this test proved that the medical interface was able to be utilized in noisy environments within a range of 10 m from medical devices. 

Many research works explored the potential of developing low-cost medical devices through the usage of Raspberry Pi and Arduino micro-controllers. Networking functionality can be included via an additional chip to enable remote access for a clinician [32]. In this example, a Wi-Fi module is applied to transmit alerts to the users in case of an emergency which in this case would be fluctuation of the readings of a sensor beyond the normal range. Wi-Fi is commonly considered less suitable for the transferal of medical data; however, Wi-Fi possesses a wider range of encryption protocols for medical device manufacturers to choose from and implement into their PHD to ensure security is upheld. Wi-Fi may require extra configuration to ensure private networks are set up as intended. Healthcare centers may wish for this level of control to mitigate the susceptibility of a medical device network. It has been convenient for medical equipment manufacturers to design Wi-Fi into a bedside and transport monitor due to the loose constraints associated with Wi-Fi power consumption and battery life [33].

The evolution of Wi-Fi has mainly been directed towards increasing networking speeds, quality of service, and wireless security. Applications with low bandwidth requirements, such as infusion pumps and patient monitoring, will properly function in the 802.11 g (2.4 GHz) and 802.11 a (5 GHz) spectrums [33]. As 802.11 n is backward-compatible with both ‘g’ and ‘a’, the same monitors will perform effectively in an 802.11 n WLAN infrastructure. Therefore, all applications can co-exist successfully on a modern WLAN network. WLANs are scalable and security and network management can be designed and deployed via VLANs. Wi-Fi is safe and reliable for patient monitoring. 

### 3.2. Web-Based Telemedicine Ventilators

To enable medical device interoperability with any other potential device to monitor it from a laptop, tablet, and mobile phone, it is necessary to include the appropriate software for each operating system (e.g., Windows, Linux, and iOS). The development of such software for every operating system would require a high level of attention. Cross-platform software development difficulties draw attention to the proficiency of a platform-independent web-based technology. Wi-Fi technologies that incorporate a universally accessible web interface offer great potential for mobile wireless devices as most of these modern devices with varying operating systems typically include a common standard for HTML document presentation. The web browser provides a favorable UI with user-friendly controls, as well as means of portraying information in multiple forms. Unifying the UI by utilizing web-based HTML lessens the development of programs for all devices. This inevitably includes its cyber security vulnerabilities due to the introduction of potentially larger network frameworks that are typically accessible throughout an entire hospital [34]. Such networks can prove troublesome and can provide malicious actors with avenues to exploit to gain access to various medical devices. Due to this, it is integral to thoroughly consider all cyber security vulnerabilities that are attached to implementing a larger network system to a life-critical device.

Telemedicine enables remote monitoring and diagnostic facilities. An automated medical monitoring system that includes decentralized supervisory control allows attention to be focused on a patient’s needs from any location. A web-based system for a medical device should embody access control. This could simply be in the form of a username and password that is required to gain access to the remote device. The users accessing the device could be any staff within the hospital, such as administrators, consultants, doctors, nurses, and students. A hierarchy of different access control levels should be implemented to limit different medical personnel accessing the medical device to certain functions [35]. Not every person who has access to the device should have full control of its function as they may not be trained to conduct activities.

In 2010, a Pulmonetic Systems LTV 1200 ventilator was modified to include a wireless interface to enable remote access through a secure wireless Internet connection [36]. A comprehensive web-based ventilator interface program was constructed and thoroughly tested to replicate the history and control screen that is portrayed locally on the ventilator. This system was applied to a simulator and was operational for many months. The prototype networked ventilator consisted of a Cellular Ventilator Monitor (CVM) that was fixated to the patient’s ventilator, along with a web server. The cellular ventilator monitor applied was a product from a reputable company, Digi International Inc, and used high-speed 3G and EDGE networks to ensure a consistent connection to the web server. Advanced Medical Electronics (AME), a company dedicated to specializing in medical devices designed software for both the CVM and the server. The software prepares data for transmission through packetization and sends it to reside on the web server. The web application enables clinicians to access this information through a web browser.

Sometimes, there is only one unidirectional link from the ventilator to the internet, not vice versa. This means that the worst-case scenario for an attack is that the medical data is exposed or manipulated in flight. The unidirectional link going from the ventilator to the remote screen should not allow control of the medical hardware to an attacker. For a WWAN, using IEEE 802.1X is critical as there is a risk of de-authorization attacks that can cause a denial-of-service condition. The main vulnerability of this network infrastructure is that the SHA-1 cryptographic hash function is employed to uphold data authenticity. This hash function has been declared fully and practically broken as a chosen prefix collision has been proven possible [37]. A trusted and unbroken cryptographic hash function should be employed for the transmission of medical device data to ensure true data privacy and to mitigate the possibility of malicious actors forging their way to accessing medical documents.

Another instance of remotely accessing mechanical ventilators describes a similar method that permits the same application infrastructure to be remotely accessed through other proprietary devices [38]. The prototype system used has been applied to Siemens Servo ventilators but supports other mechanical ventilator models such as the Puritan-Bennett 7200 AE. This remotely managed ventilator system consists of a user interface and Ethernet-based local area connections. The serial ports on the ventilator enable data to be extracted from the ventilator for processing. In this example, authenticated clinicians are given the option to remotely access telemetry results through a web-based application. The ventilator transmits data to a communications manager through a query and response approach. Every query that is packaged by the communications manager and sent through the serial interface gets redirected back to the monitoring system for clinicians. The user can interact with the system to query for particular requests. Furthermore, the monitoring system provides the capacity to register new users, automatically set notifications and alerts, and update the user log database [38].

More relevant research has been conducted to design and develop a low-cost remote infusion monitoring system. The inexpensive, Arduino compatible Wi-Fi ESP8266 microchip is included in designs to grant wireless capability to the infusion device. This research proposes two types of wireless security mechanisms to mitigate cyber threats [39]. The first one detailed is Wi-Fi Protected Access (WPA) for wireless data encryption. This protocol implements much of the IEEE 802.11i standard. WPA includes a Message Integrity Check that is designed to prevent an attacker from altering and re-transmitting data packets. This replaces the cyclic redundancy check (CRC) that was employed by the WEP standard. The second one is Media Access Control (MAC) address filtering that is set on the Wi-Fi access point. This ensures that only certain devices that have registered MAC addresses can access the network. To reduce the cost of this implementation, the system is designed to make use of a computer located at a nearby station. The assumption is that this station will have a Wireless Local Area Network (WLAN) adapter and the monitor of the computer will be used to display the data [39].

### 3.3. Medical Device Cyber Vulnerabilities and Security Controls

Researchers in [24] depicted the incline in medical device vulnerabilities between the years 1999 and 2018. These vulnerabilities are (i) improper credential management and authentication, (ii) improper access control, privilege management and authorization, (iii) stack and buffer overflow, (iv) path traversal, (v) improper input validation, (vi) information exposure, (vii) cross-site request forgery, (viii) cross-site scripting, (ix) uncontrolled resource consumption, and (x) missing encryption of sensitive data. This is persistent with the increase in the overall number of vulnerabilities reported to the CVE database (three times increase since 2013, reaching 16,555 in 2018). Furthermore, recently, CVE-2021-27410 is discovered where it may result in corruption of data or code execution on the Welch Allyn medical device management tools, whereas CVE-2021-27408 can cause information leakage leading to arbitrary code execution [40]. 

The work detailed the common categories of vulnerabilities documented by both the CVE database and ICS-CERT. These vulnerabilities include improper credential management and authentication (8%), improper access control, privilege management, and authorization (6%), and buffer and stack overflows (6%). The reported vulnerabilities had an impact on a wide range of medical devices in ranging medical specialties that were produced by 56 different manufacturing companies. It was found that 18 (12.8%) of the vulnerabilities were publicly available, conceivably allowing attackers to exploit the medical devices and affect patient safety and privacy. These vulnerabilities existed in imaging systems, hospital communication technology (for storage and communication of patient information), insulin pump or infusion pump systems, software for data and network management, and communication devices. Human misconfiguration accounts for a large portion of these vulnerabilities. Improper access control can provide particular hospital workers with access to features that they may not be trained for or authorized to conduct. Vulnerabilities that include missing encryption, cross-site scripting, and information exposure are significant as it is expected that medical device manufacturers (MDMs) and health care delivery organizations (HDOs) account for this in production or include patches to ensure this type of vulnerability is eradicated.

As described in an IEEE personal health device cyber security white paper, each security aspect relating to these devices possesses strong mitigation techniques to address such vulnerabilities. Table 2 provides a list of mitigation strategies grouped into categories defined by the NIST Cybersecurity Framework [41]. This comprehensive table of mitigation techniques should be carefully considered by MDMs and HDOs to ensure appropriate safeguards are in place.

## 4. Our Approach

This work takes a qualitative approach to perform and analyze cyber-attacks on a remote ventilator model to determine what a malicious actor could hypothetically achieve on an operational, network-enabled ventilator system used within hospitals. The model used is an Arduino-based implementation of a low-cost remotely accessible ventilator system. The makeshift implementation performs the basic functions of a ventilator. Any computer situated on the same network can access the login portal to the ventilator dashboard that controls the Arduino ventilator model. This work emanated through noticing a rise in COVID-19 related articles where the cyber security robustness of rapidly deployed, operational medical devices was challenged. The ventilator systems detailed in various articles boasted cheap production costs and utilized off-the-shelf components for fast deployment. Little consideration is taken to understand the cyber security vulnerabilities that may reside within these systems. The pandemic invoked a cohort of UK-based businesses, formally known as the VentilatorChallengeUK consortium, to collectively focus their efforts on devising low-cost ventilators to satisfy the nation’s rising demand for ventilator machines. 

Contributing companies and individuals occupy their interpretations on how a ventilator should look and what technological advancements it should include—if any. The synergization of low-cost microchips and including technological capabilities such as being able to remotely access a ventilator poses cyber concerns for the systems. It is unknown whether these microchips are adaptable enough to include additional security modifications and features to protect any data held or transmitted to or from the device. A large amount of the rapidly manufactured ventilators produced are highly mechanical and show no signs of technological advancement. However, with reports of researchers attempting to implement network capability into ventilators during COVID-19, it is not ruled out as a possibility to one of these low-cost adaptations.

All functions of the NodeMCU IoT microchip were programmed within the Arduino IDE. To provide the NodeMCU with network access, the SSID and password of a router are required. All additional components being the OLED display, air pressure sensor, and buzzer were configured within the IDE and uploaded to the physical platform through wired transmission. It was necessary to have the option to revisit and adjust the ventilator code at any time. The STRIDE risk assessment model was applied within this study to provide a systematic and structured way to identify and mitigate security risks in the ventilator web application. Utilizing this risk assessment model helped decipher what the assets were in the target system, where the vulnerabilities in the system resided, and what cyber threats could leverage the vulnerabilities if not acknowledged.

## 5. Experimental Design and Implementation

The low-cost ventilator prototype includes only two functions that can be performed remotely (i.e., being to view and alter the parameters on the ventilator). 

### 5.1. Assumptions

As of today, infrastructure utilized for securing internet connections relies on X.509 and Public Key Infrastructure (PKI) that is controlled by Certificate Authorities (CAs). The application that implements this is responsible for implementing the certificate properly. If a validation scheme is not employed, devices face the risk of being susceptible to a Man-in-the-Middle (MITM) attack. Such attacks can have significant implications specifically in the medical sector. This expresses the importance of ensuring a secure connection. Research has shown that HTTPS protection can include cache memory issues and can excessively consume server processing time. Due to this, companies fail to implement correct certificate validation in their implementations and therefore fail to secure network communication [42]. Since this work explores more on the impact of this attack, we have assumed that no strong and sufficient security level is provided for communicating over the network.

### 5.2. Designing System Model and Experiments

**Figure 2:**
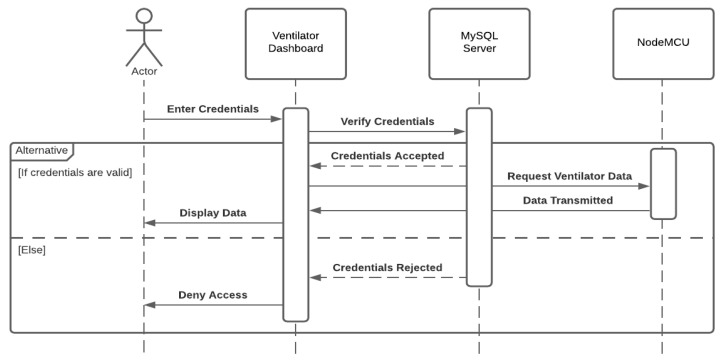
Normal scenario—communicating with a ventilator.

**Figure 3:**
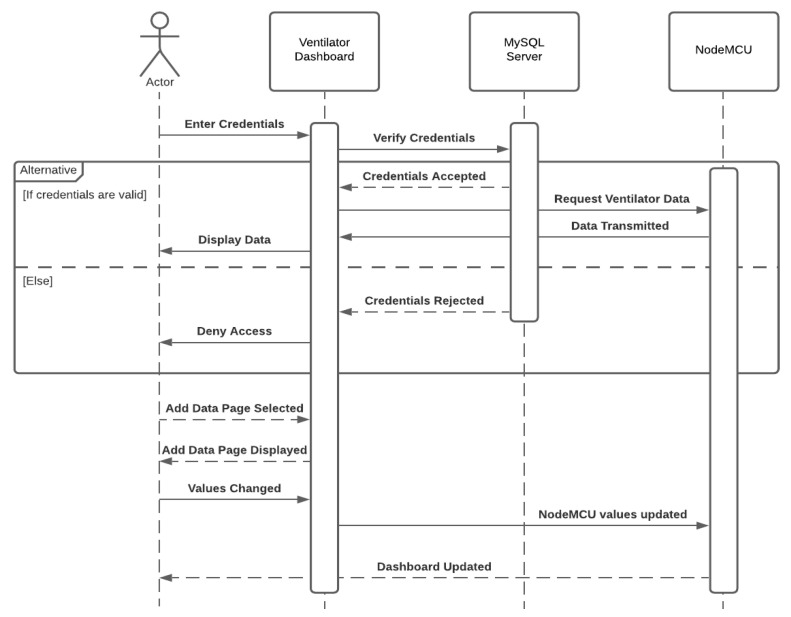
Attack scenario—data manipulating over communication with a ventilator.

Figure 2 and Figure 3 demonstrate the flow of both remote activities, one with the normal operation when accessing the ventilator remotely and another with an attack scenario manipulating data over the communication when accessing the ventilator remotely. This authorization control method was included in the Polish remote ventilator instance on which this work is based [25].

If the inserted credentials are approved by the MySQL database, the dashboard will request the NodeMCU microchip asking for the ventilator data. If the credentials are incorrect, access to the dashboard will be denied to the user and no connection is initiated between the dashboard and NodeMCU. An external server is used for storing the approved credentials for the ventilator dashboard.

The only security feature detailed in their implementation was the login portal to prevent unauthorized users from accessing the dashboard. Once the user has provided valid credentials, complete access is granted. A user can modify and submit the ventilator values and the OLED screen connected to the NodeMCU model will update to reflect the same values.

**Physical Components:** Physical components are required for this work to simulate the basic actions of a remotely accessible ventilator.

***NodeMCU:*** This microchip runs on the ESP8266 Wi-Fi SoC from Espressif Systems and includes hardware established on the ESP-12 module. The chip includes a full TCP/IP stack and micro-controller capability. This provided wireless communication between the local web server and the simulated ventilator.

***MPX4250AP:*** This component is designed to sense absolute air pressure through the intake manifold. This was included to replicate and display a patient exhales as an example ventilator parameter. A constant stream of real-time data from this component is transmitted to the web server to provide the simulation of constant patient monitoring.

***OLED I2C Display:*** This display was used to confirm that the values entered and updated from the web portal had also been updated on the board.

***Speaker Buzzer:*** This was utilized as a warning. The speaker would sound if particular values for editable ventilator elements were outside of their recommended boundaries.

***Raspberry Pi 4 Model:*** This was used for hosting the MySQL server that stored user credentials. User credentials were required to access the ventilator dashboard to assure only authorized users can modify the ventilator controls.

**Figure 4:**
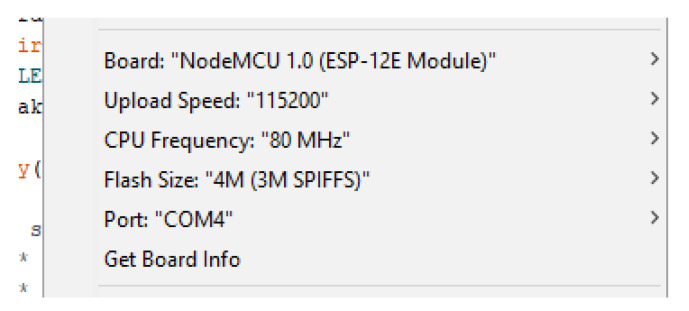
Arduino parameter settings used.

A keyboard, mouse, Micro HDMI to HDMI cable, USB Type-C to USB-A 2.0 cable, and a Micro USB cable were used for providing power to the microchips and uploading code. To upload the C++ ventilator code to the NodeMCU, the settings shown in Figure 4 are used.

**Experiment Setup:** These components are cheap, easily accessible, and previous related studies make use of them to develop low-cost medical devices. Every component excluding the Raspberry Pi was attained to replicate certain individual elements on an operational ventilator. It is understood that this model may not be the most advanced replica to work with; however, being able to access the NodeMCU remotely within a LAN does simulate a low-level instance of network-enabled ventilators. These ramifications are due to the time constraints and budget associated.

**Figure 5:**
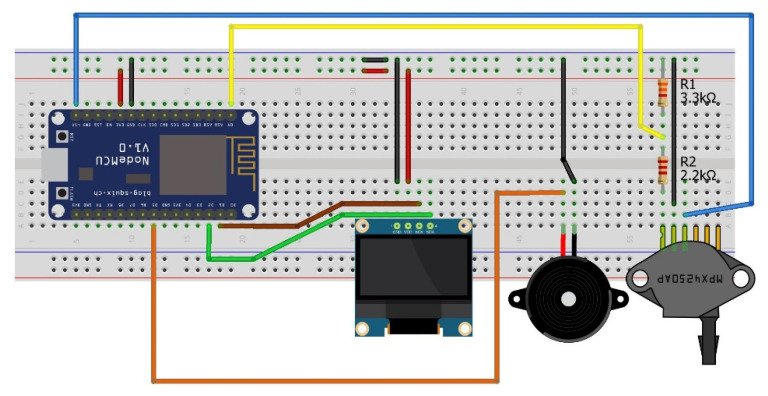
NodeMCU connections.

Figure 5 shows the finalized breadboard setup for the model. This shows the wiring associated with each component and how they are connected to the core NodeMCU. The wiring convention used is as follows: Red is for live, black for ground, and every other color is how each component connects to the NodeMCU. Resistors are used for the MPX4250AP air pressure valve to limit the voltage sent to it as, otherwise, the component would break.

An amalgamation of open-source software tools is required and needs installing to create the web server. MariaDB, a MySQL relational database management system is required for installation on the Raspberry Pi to create a database to handle authorization control for the ventilator dashboard. Once created, PHPMyAdmin, an open-source administration tool for MySQL and MariaDB will be employed to enter credentials accepted by the ventilator dashboard as it provides a simple GUI interface as opposed to solely using the command line. As for the website, Angular 8, an open-source web application framework will enable fast deployment of the website dashboard environment. To make the website live, Apache2, an open-source cross-platform web server software will be executed as a background service to host the website. Both the MySQL database and the ventilator dashboard will execute on the Raspberry Pi to provide these services with their own external space. The Raspberry Pi will be allocated its IP address that through the LAN, can be accessed by another computer. Any computer within the network will be able to locate the IP of the Raspberry Pi and access the log-in portal for the NodeMCU ventilator dashboard.

### 5.3. Implementation and Experiment Execution

**Figure 6:**
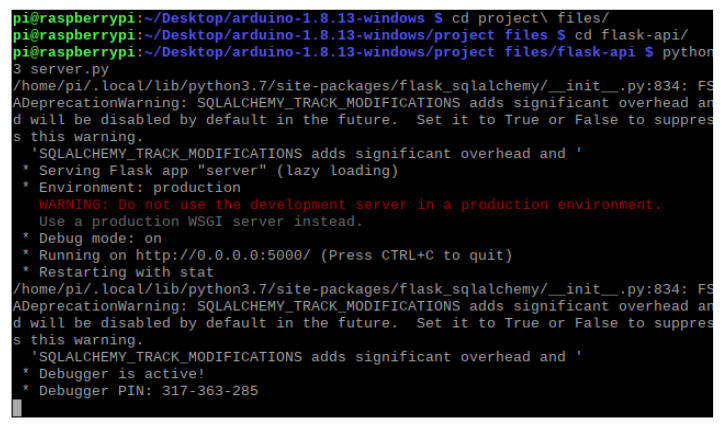
Executing the server.py script on the Raspberry Pi.

**Figure 7:**
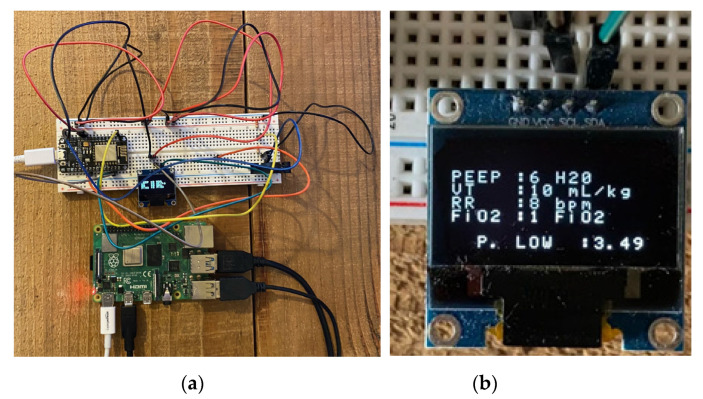
(**a**) NodeMCU ventilator implementation and Raspberry Pi, (**b**) value inserted on dashboard updated on NodeMCU.

To start the web server, a Python script must be manually executed on the Raspberry Pi. This script includes imports from SQLAlchemy, a library that facilitates the communication between Python programs and databases, and Flask, a micro web framework written in Python. This allows for the credentials entered into the login portal to be validated against the MySQL database containing approved user credentials. Figure 6 shows the script is successfully executed on the Raspberry Pi. This Arduino-based model allows for this to be demonstrated as bidirectional data is transmitted between the host computer running the dashboard and the NodeMCU. This information can be intercepted and can be leveraged by an attacker. Figure 7a shows the implementation of the low-cost ventilator model and the Raspberry Pi. The wiring configuration of the NodeMCU is disorganized as longer wires had to be purchased for the model. This was due to issues with the original wires being faulty and not properly connecting to the OLED screen. Figure 7b shows that the values displayed on the OLED reflect the new values injected into the ventilator dashboard.

**Figure 8:**
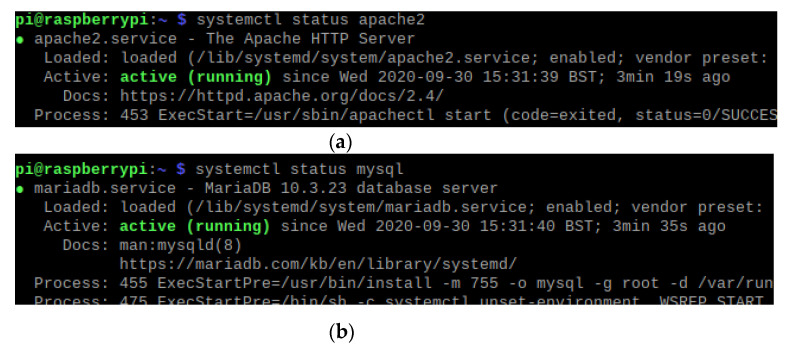
(**a**) MySQL service running on Raspberry Pi. (**b**) Apache2 service running on Raspberry Pi.

To ensure both the MySQL and Apache2 services are running on the Raspberry Pi the Unix command systemctl status was used. As shown in Figure 8a,b, both services are installed correctly and are active.

**Figure 9:**
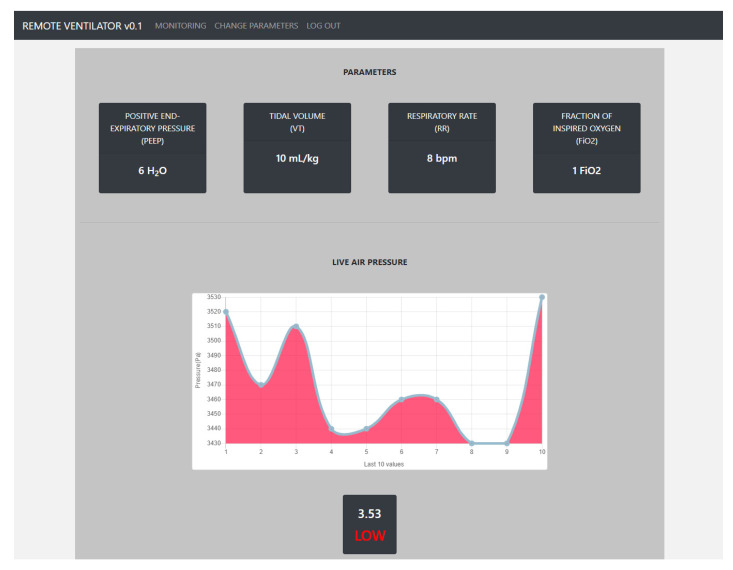
Remote ventilator dashboard reflecting the change in parameters over the communication network.

Once the services were running as intended, the focus was on monitoring the dashboard to observe the reflected changes, as shown in Figure 9. To be able to develop the Angular, the Angular CLI package and other Node.js dependencies were required to build the web interface prior to being able to upload it to the Raspberry Pi. 

These features with ventilator machines are the Positive End-Expiratory Pressure (PEEP), Tidal Volume (TV), Respiratory Rate (RR), and the Fraction of Inspired Oxygen (FiO_2_). These values are observed on the NodeMCU ventilator model and are used to expose the cyber security vulnerabilities associated with the transmission of these values over a wireless network. The live air pressure records the last 10 values and formats the data into a real-time updated graph. Authorized users can update the ventilator parameters with new values such as positive end-expiratory pressure (PEEP), total volume (VT), respiratory rate (RR), and a fraction of inspired oxygen (FiO2). These new values will be transmitted via the LAN to the NodeMCU module. Upon inserting new values and pressing submit, a notice will appear at the top of the page to either confirm or reject the request. The main focus of the work revolves around how a perpetrator can access these values in flight using packet sniffing. 

### 5.4. Attack Scenarios

**Figure 10:**
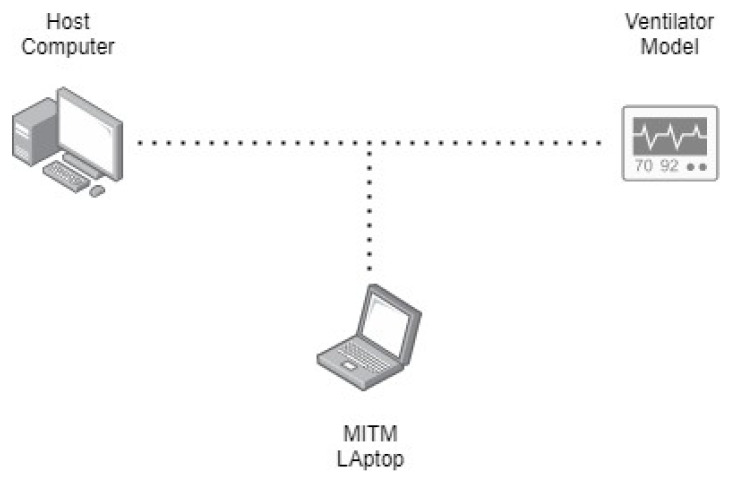
Man-in-the-middle attack performed.

The attack simulation focuses on packet sniffing to illicitly unveil unencrypted sensitive information in transmission. Wireshark is utilized on a separate device to capture data packets being sent from the computer running the ventilator dashboard to the NodeMCU ventilator implementation. The separate machine is given access to the same network in which the medical device simulation is operative. The external device listening into the network is used to replicate how a malicious actor can use packet sniffing to access sensitive data transmitting unencrypted across a network as shown in Figure 10. The interfering machine is provided network access to the medical device LAN are hospitals being public establishments and any adversary who intends on performing such an attack in a real-world scenario.

The Positive End-Expiratory Pressure (PEEP), Tidal Volume (TV), Respiratory Rate (RR), and the Fraction of Inspired Oxygen (FiO_2_) integer values used on the ventilator model can be adjusted from a remote location through the use of the web dashboard. This experiment captures and modifies the contents of the data packets that accommodate the raw, unencrypted values sent to the NodeMCU ventilator model. The repercussions of a malicious actor viewing and modifying these values are detrimental and in a real word scenario, the third-party interference could kill a patient. This type of attack can be performed in various locations and go undetected if values are only altered by an unnoticeable amount.

#### 5.4.1. Packet Analysis with Normal Scenario

**Figure 11:**
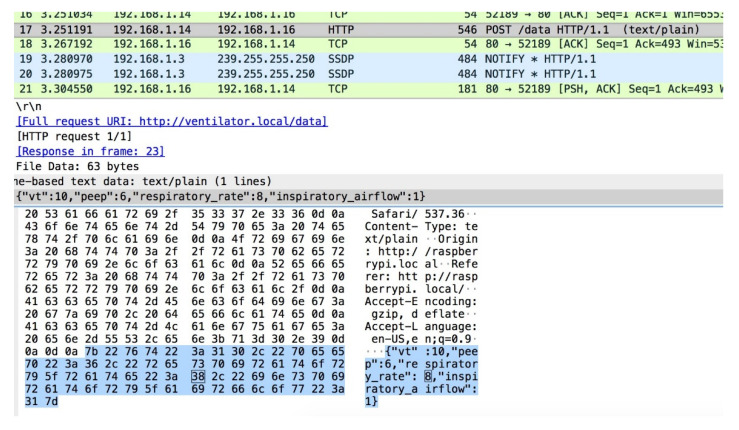
The capture of values under the normal scenario.

As shown in Figure 11, Wireshark displays the HTTP POST request sent from the ventilator/user interface to the NodeMCU ventilator model. To initiate the POST request, the machine hosting the ventilator dashboard updated the ventilator model controls with new values. These values were then intercepted in transmission using another laptop posing as a malicious actor. A malicious actor can modify these values to adjust how the ventilator model functions. These values are not as frequent on the network and will only be visible when an authorized user updates the ventilator with new values.

**Figure 12:**
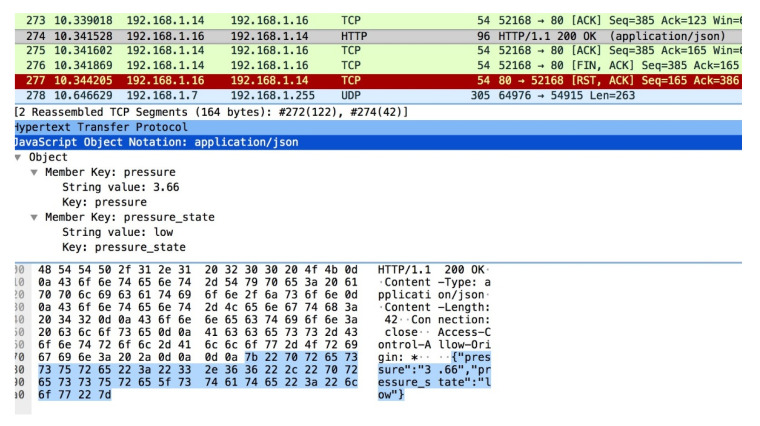
Capture adversarial sensor values under attack scenario.

Figure 12 displays the HTTP GET request sent from the adversary dashboard to the NodeMCU ventilator model. This capture requests for the NodeMCU ventilator model to send the sensor data. This data is more persistent on the network as it constantly updates the dashboard with a real-time pressure reading. The air pressure sensor value can be intercepted and modified in flight. Doing so and displaying an incorrect measurement on the dashboard may influence a clinician to update particular ventilator values to account for the counterfeit reading. The updated values could have a detrimental effect on the patient’s lungs and, in the worst-case scenario, could potentially kill the patient.

#### 5.4.2. Packet Manipulation

To exploit the ventilator network and demonstrate this attack, a packet manipulation tool Scapy is employed. Scapy is a Python program that can handle a wide range of networking tasks such as scanning, trace routing, probing, unit testing, performing attacks, or discovering networks. This software tool used in parallel with Wireshark can allow an attacker to manipulate how the remote ventilator model functions. The initial stage of locating a particular medical device on a network can be challenging; however, once the IP of a device has been located using Wireshark, fake packets can be generated using Scapy to replicate genuine packets. Scapy enables an attacker to spoof the source of the packet and allows packets to be sent to a particular address through simple command-line usage. To send counterfeit packets to the NodeMCU, the attacker would have to simulate the TCP three-way handshake to send a POST request. Once a connection has been established with the server running the web interface, counterfeit packets crafted using the same key and value structure used in the legitimate packets can be injected into the NodeMCU to override the set values for the ventilator controls. It is important to do this within the active session, otherwise, the security key that was captured may not be valid any longer. The Python Scapy snippet beneath shows how an SYN packet can be crafted in the command line. This sends the IP ‘192.168.1.16’ an SYN request. This is the IP for the ventilator server.


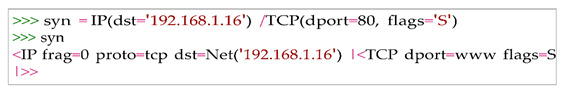


Once the SYN-ACK has been received from the server, the HTTP POST request can be sent as shown beneath.


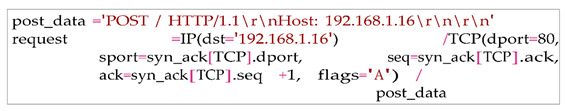


Values can be transmitted to the ventilator server using this method by replicating the POST request structure seen in Wireshark. 

#### 5.4.3. Attack Repercussions

Each remotely adjustable ventilator function has boundaries delineating what each value can be safely updated to. Some of the ventilators’ controls when set at particular values conflict and cannot function concurrently as they are invoked in different conditions. As an example, when the FiO_2_ is set to 50% or less, most clinicians will select a PEEP of 5 to 8 cm H_2_O. Whereas when the FiO_2_ is set above 50%, clinicians will make use of a higher PEEP such as 10 cm H_2_O. A breakdown of the conventional boundaries for each control is specified in Table 3.

The ventilator should only deliver breaths at the specified respiratory rate set by an authorized clinician. The only exemption occurs when a patient breathes faster, thus triggering the ventilator to assist at a faster rate. Ventilator functions that deviate from their typical settings carry potential consequences including pneumothorax, airway injury, alveolar damage, ventilator-associated pneumonia, and ventilator-associated tracheobronchitis. An experienced clinician may notice that a ventilator control has been tampered with if their values do not correlate with the typical alleviation values being used by the clinician for a particular ventilator setting. 

**Figure 13:**
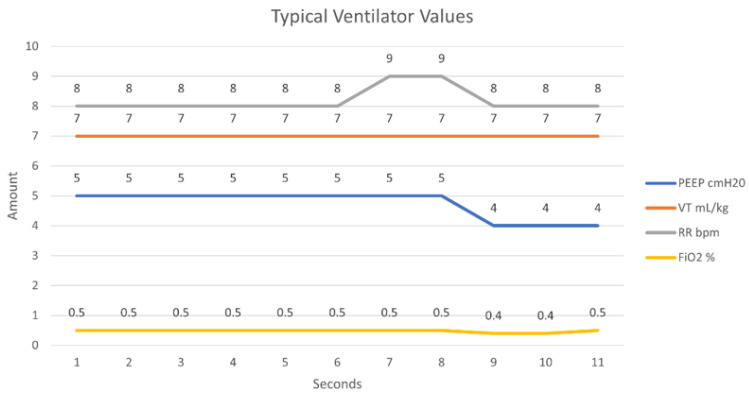
Simulated ventilator parameters under the normal scenario.

**Figure 14:**
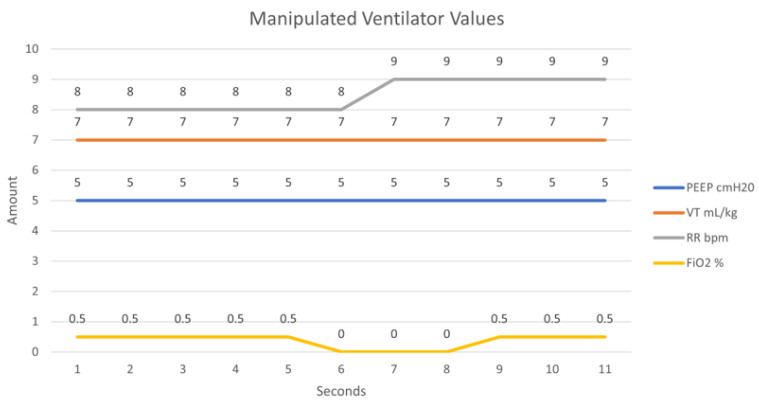
Simulated ventilator parameters under attack scenario.

Figure 13 exhibits what the values used in this experiment would typically be set as on an operational ventilator under normal operation (scenario). A malicious actor (adversary) who has successfully eavesdropped onto the network can transmit counterfeit packets to the remote ventilator to adjust its performance. Figure 14 illustrates an attack difficult to notice whereby the rate of FiO_2_ is set to 0 for a short duration of time. The FiO_2_ value controls the concentration of oxygen in the ventilator gas mixture. Setting this value at 0 for a brief duration would deprive a patient of their oxygen. This attack carries extreme consequences for a patient and if done subtly, can be undetected by a clinician unless an alarming system is used on the ventilator system to signal such occurrences.

A more blatant attack can be performed by an adversary whereby the ventilator values are overridden with new values fair from their typical boundaries. As an example, increasing the PEEP to an unusually high value would cause the alveoli to open and collapse much more than usual. This would lead to serious alveoli damage to a patient. Similarly, increasing the FiO_2_ value too high could also affect alveolar ventilation, reverse hypoxic vasoconstriction, induce pulmonary toxicity, and can reduce tissue blood flow due to vasoconstriction. These sporadic changes are more noticeable but can invoke a greater amount of damage to a patient.

## 6. Discussion on Attack Impact and its Mitigation

The low-cost NodeMCU chip employed as the core of the ventilator model had limitations on what IoT encryption protocols could be included. Enabling encryption on the NodeMCU requires some effort and the firmware compiled needs to include the cryptography module within the application. The ESP8266 NodeMCU does not offer any real-time flash encryption capability or code signing (hence, straightforward to target an attack!) and because of this, it is difficult to secure the platform. Despite this, it is still possible to create a system where encrypted and authenticated messages can be transmitted using an MQTT server to either an ESP8266 or a larger system running Python using AES-CBC mode with HMAC encryption. MQTT is an open OASIS and ISO standard lightweight, a publish-subscribe network protocol that transports messages between devices over the TCP/IP layer. The keys generated are stored in the flash memory of the NodeMCU. This requires access control to ensure reverse engineering of the keys is not possible. Many flaws have been identified in the ESP microchip family and patches have been provided. One of the simplest exploits unearthed relates to over-flowing the NodeMCU access point buffer. When the NodeMCU connects to an access point, an AKM suite count is sent from the access point that lists the number of authentication methods available for the connection to be established. A malicious fake access point posing as one of the hospital’s access points can send a large number to attempt to overflow a buffer as the NodeMCU typically does not perform bounds-checking on this value. If it is possible to send a bogus beacon frame or a probe response, it can be rendered unresponsive. The patches provided have supposedly fixed this security vulnerability; however, there are millions of these microchips that are in constant use that have potentially not updated their firmware. These experiments intended to express the importance and repercussions associated with medical device data transmitting in plain text across a network in light of the COVID-19. The scenario created has been done so to emulate the remotely accessible ventilator model operational in Poland whereby a ventilator could be controlled using a web interface from any location within the hospital.

***Mitigation:*** To mitigate this attack, only trusted internal IPs associated with hospital computers should be able to make a connection with the server. A clinician who makes use of a remote system will typically be located in another room and will not be bedside to visually observe a patient’s condition. Due to this, it could extend the time taken to discover the declining condition of a patient. This is why it is imperative to implement alarms to alert a member of staff when a patient is experiencing discomfort. These signals are typically found on the machines themselves. If similar alarms were to exist on the ventilator dashboard, they could also be exposed to modification and may be reconfigured to become inaudible or to not display by using similar networking techniques. This could cause a wide range of complications for hospital workers who are unknowing of why the remote ventilator is malfunctioning. One preventative measure that can be taken about the security of the website could be to make use of HTTPS. This would encrypt the data by using the Transport Layer Security protocol, rendering packets transmitted across the network unreadable to an unintended recipient. It is important to consider the software used on the NodeMCU as a wide number of attacks occur due to insecure software. The security of the ventilator device is not solely based on running HTTPS. As well as this, there is the possibility that the host computer used to view the web dashboard is infected with spyware and someone is reading the credentials entered into the dashboard portal. It is anticipated that the operational implementation in Poland incorporates sufficient cyber security previsions and only certified devices that are strictly used within the hospital can use the dashboard.

## 7. Conclusions and Future Work

This study focuses on medical device networks based on the Wi-Fi network protocol. A wide range of studies discussed considers other protocols that are applied in the medical sector such as Zigbee and Bluetooth. These transmission protocols each have their benefits and drawbacks on how they would suit this experiment; however, Wi-Fi was surmounted as the chosen protocol as the scenario this work was based on Wi-Fi connecting to a remote ventilator. The motive behind this study was to express the importance of stringent cyber security for all medical devices that possess networking capabilities. This is especially important amidst COVID-19 as alternative methods have been applied across the world in an attempt to overcome the shortage of ventilator machines. The standards released by the MHRA delineate the considerations that companies need to address when building an RMVS machine. There is little documentation on the cyber considerations in which a company has to acknowledge when constructing these devices. It is understood that the companies developing these systems strive for innovativeness and implementing technological capabilities into one of their ventilator models is a possibility.

An in-depth overview of the cyber secureness of networked medical devices and hospitals was explored in this work. A wide range of studies discussed considers other protocols that are applied in the medical sector with their benefits and drawbacks to reflect on gaps. The motive behind this study was to express the importance of stringent cyber security for all medical devices that possess networking capabilities. This is especially important amidst COVID-19 as alternative methods have been applied across the world in an attempt to overcome the shortage of ventilator machines. The standards released by the MHRA delineate the considerations that companies need to address when building an RMVS machine. There is little documentation on the cyber considerations in which a company has to acknowledge when constructing these devices. It is understood that these rapidly deployed ventilator machines are very mechanical and do not require many components. However, the companies developing these systems strive for innovativeness and implementing technological capabilities into one of their ventilator models is a possibility.

A simulation including low-cost microchips was developed to simulate bidirectional being sent from both the ventilator dashboard and the ventilator model. This model was used to express how the data transmitted to and from the ventilator model can be intercepted and maliciously leveraged if not encrypted. The repercussions of this were detailed to express the severity of such information is readily available. The ESP8266 NodeMCU chip that ran the entirety of the ventilator model provided few security features. It was discovered when looking for encryption implementations that the microchip had been exposed to a variety of network-based attacks and patches for the component had to be released to cater for its vulnerabilities. An attempt to contact the companies working on producing the rapidly deployed ventilator systems was made, although, due to time constraints, this could not be continued, which we now plan for future work.

This study is topical and is of great interest amidst a pandemic. This work required primary research from one of the companies developing the rapidly manufactured ventilators. To accurately test and document the vulnerabilities associated with being able to remotely access a ventilator, a realistic setup is required. It is known that the companies producing these machines are technology-led and pride themselves on their pioneering efforts; however, including these capabilities into medical devices may include vulnerabilities that otherwise the device would not have. To expand on this in the future, it would be beneficial to make use of a wider array of attacks to attempt to override the login portal and exploit other areas of the system. An in-depth overview of a remotely accessible prototype provided by an external company that details its vulnerabilities and provides mitigation strategies to the vulnerabilities would help broaden this area of study and would expose the importance of securing networked medical devices that transmit data across potentially accessible networks.

## Figures and Tables

**Table 1:** Comparison between ZigBee and Bluetooth Low Energy (v4.2) BLE.

Characteristic	ZigBee	Bluetooth Low Energy
IEEE Specs	802.15.4	80.15.1
Range	10–100 m	Greater than 100 m
Data rate	1–3 Mbps	1 Mbps
Power profile	Low	Low
Operating frequency	868/915 MHz, 2.5 GHz	2.4 GHz
Security	128-bit AES plus application layer security	128-bit AES with Counter Mode CBC-MAC
Robustness	16-bit CRC	24-bit CRC, 32-bit Message Integrity Check
Network Topology	Star, mesh, cluster tree	Star, point-to-point
Application Focus	Monitoring and control	Cable replacement

**Table 2:** Mitigation categories, security capabilities, and mitigation techniques.

Mitigation Category		Security Capability	Mitigation Technique
Identify		Node Authentication	Authentication
		Personal Authentication	Digital Signatures
Protect	Protect-Prevent	Authorization Health Data De-identification Health Data Storage and Confidentiality Health Data Integrity and Authenticity Physical Locks on Devices Automatic Logoff	Authorization De-Identification Do not Store Secrets Encryption Filtering Message Authentication Code
	Protect-Limit	Configuration of Security Features Software and Application Hardening Security Guidelines	Physical Tamper Resistant Input Sanitization Input Validation Quality of Service Least Privileges Throttling
Detect		Audit	Audit Trail
		Physical Locks on Devices	Physical Tamper Evidence
Respond		Malware Detection and Protection	End-User Signalization
		Emergency Access	Invalidate Compromised Security
Recover		Data Backup and Disaster Recovery	Re-Establish Security
		Cybersecurity Product Updates	

**Table 3:** The typical boundaries for mechanical ventilator controls.

Ventilator Parameters	Typical Range	Description
Positive End Expiratory Pressure (PEEP)	5–10 cm H_2_O	A small amount of PEEP (5 cm H_2_O) is typically applied in most mechanically ventilated patients to mitigate end-expiratory alveolar collapse. A higher level of applied PEEP (above 5 cm H_2_O) is used to improve hypoxemia or reduce ventilator-associated lung injury in patients with acute lung injury, acute respiratory distress syndrome, and other types of hypoxemic respiratory failure.
Tidal Volume (VT)	6–15 mL/kg of body weight	Historically, initial tidal volumes have been set at 10 to 15 mL/kg of the body weight of patients with neuromuscular diseases. The low-tidal-volume strategy uses 6 mL/kg of predicted body weight and has become the standard of care for patients with ARDS. Tidal volumes greater than 10 mL/kg of body weight are not routinely applied.
Respiratory Rate (RR)	3–12 bpm	A respiratory rate (RR) of 8 to 12 breaths per minute is endorsed for patients not requiring hyperventilation for the treatment of toxic or metabolic acidosis, or intracranial injury. Initial rates for asthmatic patients may be as low as 5–6 breaths per minute to use a permissive hypercapnic technique.
Fraction of Inspired Oxygen (FiO_2_)	0.5 and below	Natural air includes 21% oxygen which equates to a FiO_2_ of 0.21. FiO_2_ is typically maintained below 0.5 even with mechanical ventilation, to avoid oxygen toxicity, but there are applications when up to 100% is routinely used.
